# Redescription of arenicolous dipluran *Parajapyx pauliani* (Diplura, Parajapygidae) and DNA barcoding analyses of *Parajapyx* from China

**DOI:** 10.3897/zookeys.221.3207

**Published:** 2012-09-13

**Authors:** Yun Bu, Yan Gao, Mikhail B. Potapov, Yun-Xia Luan

**Affiliations:** 1 Institute of Plant Physiology & Ecology, Shanghai Institutes for Biological Sciences, Chinese Academy of Sciences, Shanghai 200032, China; 2 Moscow State Pedagogical University, Kibalchich str., 6, korp. 5, Moscow 129278, Russia

**Keywords:** Diplura, *Parajapyx*, littoral, DNA barcodes analysis, China

## Abstract

Littoral dipluran *Parajapyx pauliani* Pagés, 1959 was redescribed based on the specimens collected in Hainan Island, South China. The littoral habitat was confirmed for the species, as the first report of arenicolous dipluran in China. DNA barcoding fragment was sequenced for five *Parajapyx* species (18 individuals) from China, and this is the first report on DNA barcodes used for dipluran identification. The mean intra- and interspecific divergencesare 1.9% and 19.1% respectively. Synonymy of *Parajapyx paucidentis* and *Parajapyx isabellae* was confirmed.

## Introduction

The genus *Parajapyx* was erected by Silvestri (1903) with type species *Parajapyx isabellae* (Grassi, 1886). It is characterized by the mandible with five teeth and four denticles, absence of labial palpus, maxilla with first lobe slender and others pectinate, two pairs of spiracles on meso- and metanotum, four placoid sensilla on the terminal segment of antenna, subcoxal organ on urosternite I, eversible vesicles on urosternites II and III, claw with single medial unguis, and symmetrical cerci with 4-5 inner teeth (Pagés 1952, Xie and Yang 1992).

Later, *Parajapyx* was divided into two subgenera (*Grassjapyx* and *Parajapyx*) according to the shape of cerci (inner margin of cerci straight, tooth 1 not separated from others by a sinus in *Grassjapyx* vs. teeth 2-5 or 3-5 on a convexity of the internal margin, tooth 1 is separated from others by a sinus in *Parajapyx*) (Pagés 1952). So far, there are 31 species (16 subspecies) described in subgenus *Grassjapyx*, and 24 species (7 subspecies) in subgenus *Parajapyx* (Sendra 2006, Luan et al. 2007) in the world. Five species of genus *Parajapyx* were reported in China (Xie and Yang 1992, Luan et al. 2007).

In April 2011, during the research of the diversity of basal hexapods in littoral of Asia-Pacific coast, seven specimens of *Parajapyx* were collected from intertidal zone of several beaches of Hainan Island, South China. Those specimens were identified as *Parajapyx pauliani* Pagés, 1959, which was firstly described based on only specimen from intertidal zone of Nosy Be, Madagascar Island, and Pagés doubted about the habitat where the species was collected (Pagés 1959).

In this study, we provided a detailed redescription of this species based on our specimens, and more discussion on its littoral habitat. We analyzed the DNA barcoding sequences (Hebert et al. 2003) of *Parajapyx pauliani*, as well as other four *Parajapyx* species living in soil, in order to confirm the validity of species, and provide a useful reference for the identification of *Parajapyx* species.

## Materials and methods

### Samples collection

With flotation method, the specimensof *Parajapyx pauliani* were collected directly from the water surface in Hainan, China, and stored in 80% ethanol. Specimens of other species were extracted by the Tullgren funnels from soil samples (Table 1). For *Parajapyx isabellae*, two individuals of its synonym *Parajapyx paucidentis* identified from the morphology were also sampled.

**Table 1.** *Parajapyx* species and outgroups used in the study.

**Classification**	**Species**	**Locality**	**Number of individuals**	**GenBank Accession Numbers**
**Diplura**				
**Parajapygidae**				
***Parajapyx***	*Parajapyx pauliani*	Hainan	2	JQ692327, JQ796634
	*Parajapyx emeryanus*	Shanghai	6	JQ796635**-**JQ796640
	*Parajapyx isabellae*	Shanghai	5	JQ796641**-**JQ796645
	*Parajapyx isabellae* (Syn. *Parajapyx paucidentis*)	Shanghai	2	JQ796646, JQ796647
	*Parajapyx hwashanensis*	Qinghai	1	JQ796648
	*Parajapyx yangi*	Gansu	2	JQ796649, JQ796650
**Japygidae**	*Occasjapyx japonicus*	Shanghai	1	HQ882833
**Campdeidae**	*Lepidocampa weberi*	Shanghai	1	HQ882832

### Taxonomy of *Parajapyx pauliani*

Seven specimens of *Parajapyx pauliani* were collected: four of which were mounted in Hoyer’s solution for identification, two were morphological identified in the alcohol first and then used for DNA extraction, and one was reserved in pure alcohol. Measurements and photos were taken by the help of a phase contrast microscope NIKON E600. The species was identified by the comparison of characters of all known species of the genus. For the name of chaetotaxy, we used the nomenclature proposed by Pagés (1952, 1996), and made some minor modifications following García-Gómez (2009). Microsetae on the body and the sensilla on the antenna were studied in detail for this species. Each pro-, meso- and metasternum was divided to three areas to designate setae.

Abbreviations. Ant. I-XXI= antenna segments I-XXI; BS= baculiform sensillum; M = macroseta; the position on dorsal of body as: ma = medial anterior, la= lateral anterior, mp = medial posterior, lp = lateral posterior; ms= microsensillum; m = microseta, n* = normal seta; s=sensillum; t1-t5= teeth of cercus.

* including all “s” setae named by Pagés (1952) and all supplemental setae inserted between M.

### Molecular experiments

Eighteen individuals from five *Parajapyx* species were used for DNA barcoding analyses (Table 1), and two dipluran specimens from Japygidae and Campodeidae were used as the outgroups. All specimens were morphological identified in the alcohol first and then used for DNA extraction. We followed the experimental procedure for Collembola described in Potapov et al. (2010). Genomic DNA was extracted from one individual using the Wizard SV Genomic DNA Purification System (# 2361). The mitochondrial COI gene sequence was amplified (658 bp) by primer pair LCO (5’ - GGTCAACAAATCATAAAGATATTGG-3’) / HCO (5’- TAAACTTCAGGGTGACCAAAAAATCA- 3’) (Folmer et al. 1994). PCR products were purified and then sequenced directly using both of the amplification primers.

### Sequences analysis

DNA sequences were analyzed with the software DNASTAR (Burland 2000). The genetic divergences (p-distance) were analyzed using MEGA 4.0 (Tamura et al. 2007). The phylogenetic tree was constructed by PAUP 4.0 beta 10 (Swofford 2002) with Neighbour-joining method and 1000 bootstrap replicates.

## Results

### Parajapyx pauliani

Pagés, 1959

http://species-id.net/wiki/Parajapyx_pauliani

Figs 1–17 Tables 2 3

#### Material examined.

4 females, South China, Hainan Island, Sanya city, shingly beach of Ximaozhou island (samples No. 6 and 8), 18°14'N, 109°22'E, 5-IV-2011; 1 female, from sand beach of the Ximaozhou Island (sample No. 17), 6-IV-2011; 2 female, South China, Hainan Island, Changjiang County, Changhua town, from sand beach of Qizi Bay (sample No. 54), 19°21'N, 108°40'E, 7-IV-2011, coll. Y. Bu, C. W. Huang, M. B. Potapov and N. A. Kuznetsova. All specimens are kept at Institute of Plant Physiology & Ecology, CAS.

#### Redescription.

**Figures 1–17.**
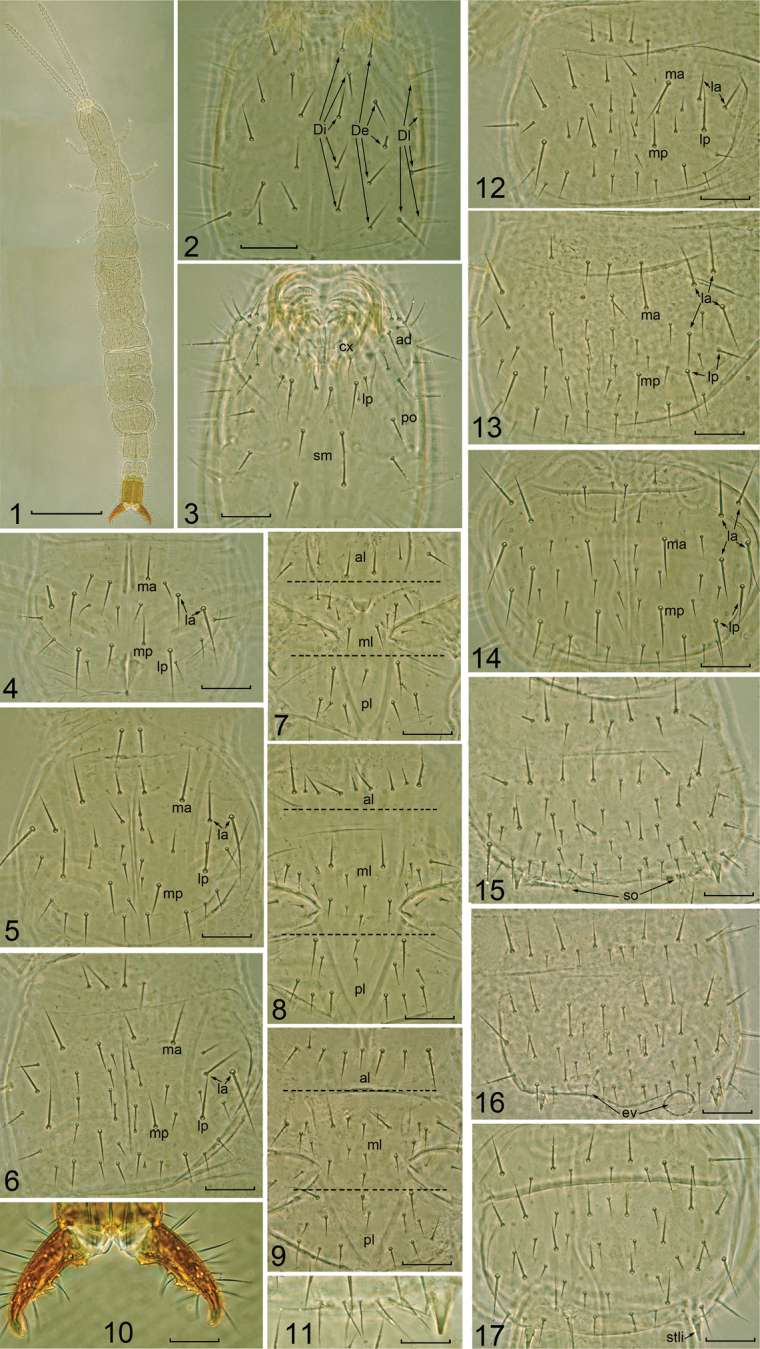
*Parajapyx pauliani* **1** Habitus **2** head, dorsal view (Di= dorsal interior setae; De= dorsal exterior setae; Dl= dorsal lateral setae) **3** head, ventral view (**ad**= admentum **cx**= coxae **lp**= labial palps area **sm**= submentum **po**= pli oral region) **4** pronotum **5** mesonotum **6** metanotum **7** prosternum (**al**= anterior lobe **ml**= middlelobe **pl**= posterior lobe, same for figs **8–9**) **8** mesosternum **9** metasternum **10** cerci **11** subcoxal organ of urosternite I, right side **12** urotergite I **13** urotergite II **14** urotergite VII **15** urosternite I (**so**= subcoxal organ) **16** urosternite II (**ev**= eversible vesicles) **17** urosternite VII. Scale bar: 0.5 mm in Fig. **1**; 0.1 mm in Figs **2–17**.

Body length and width of adult female 2.8–3.0 mm, and 0.3–0.35 mm, respectively (four specimens, antenna and cerci not included). Tegument smooth, without ornamentation (Fig. 1).

#### Head. 

Length 0.23–0.25 mm, width 0.23–0.25 mm. Dorsal side with 5+5 interior (Di), 5+5 exterior (De), and 10+10 lateral setae (Dl) (only show five on the picture), without front setae (Fig. 2). Labrum with two pairs of medial setae (1+1 M and 1+1 n), 6+6 m. On ventral side internal lobe (li) with 1+1 m; external lobe (le) with 9 +9 setae; coxae (cx) with 1 M and 3n; labial palpus absent, replaced with 1 M accompanied by two normal seate; admentum with 11 setae, 3 M and 8 n; pli oral region with 4–5 setae; submentum with 2+2 setae (Fig. 3).

Mouthparts. Lacinia composed by five lobes, the first lobe (distal) is very acute and smooth, and the following four larger and pectinate. Mandible with five teeth and three denticles between them. Maxillary palpus with 10 n and 2 m setae.

Antenna with 21 segments, length 0.8 mm. Antenna segment I with seven microsetae dorsally and 5 setae, Ant. II and III each with 9 setae, Ant. IV with 11 setae, Ant. I-IV without sensilla and trichobothria, Ant. V with two bacilliform sensilla (BS) and 14–17 setae, Ant. VI with three BS and 16–18 setae, Ant. VII with three BS and 17–20 setae; Ant. VIII-XIX each with 4 BS and 18–28 setae, Ant. XX with 6 BS and 34–35 setae, Ant. XXI with eight BS and four placoid sensilla, and 55–60 seate. Single microsensillum asymmetrically present on Ant. IX-XIII, XVII, and XIX.

**Thorax.** Chaetotaxy of thorax as show in Table 2, 3. Pro-, meso- and metanotum each with 5+5 M setae and 6-17 n setae (Figs 4–6). Pro-, meso- and metasternum as show in Figs 7–9. Leg III length 0.3 mm, coxa with 1 M, 3 n and 2 m; trochanter with 1 M and 2 n dorsally, 1 m ventrally; femur with 10 n and 3 m setae; tibia with 8 n; tarsus with 10 n; claw symmetrical and with single medial unguis.

**Abdomen.** Chaetotaxy of the abdomen as shown in Table 2 and 3. Urotergite I (Fig. 12): prescutum with 4+4 m and 2+2 n, scutum with 6+6 m, 5+5 M and (10-14)+(9-14) n. Urotergites II-VII (Figs 13-14): prescutum with 4+4 m and 2+2 n, scutum with (6-8)+(6-8) m, 8+8 M and (9-18)+(9-19) n. Urotergite VIII with 12+12 m, 8+8 M and (9-10)+2(3)+(7-13) n. Urotergite IX with 7+7 m, 3+3 M and 2+1+2 n. Urotergite X with 4+4 m, 6+1+6 M and (6-8)+(6-8) n.

Urosternite I (Fig. 15): prescutum with 2+2 m and 5 +5 n, scutum with 5+5 m, 10+10 M and (10-18)+1(2)(3)+(11-18) n. Subcoxal organ composed by 10-13 setae, including 6-8 slender glandular setae and 4-5 sensory setae, without medial glandular organ (Fig. 11). Urosternites II to VII (Figs 16-17): prescutum with 4 +4 m and (5-7)+1+(5-7) n setae, scutum with 5 +5 m, 12 +12 M, (7-12) + 2(3)+(6-11) n. Urosternite VIII with 4+4 m, 2 +2 M, (4-5)+(4-5) n. Urosternite IX with 4+4 m, 2 +2 M and 3+3 n. Urosternite X with 4 +4 m, 6+6 M and 3+1+3 n. Eversible vesicles present on the urosternites II-III, diameter 32-37 μm. Styli on urosternites I-III each with one short sensilla and one m seta, on urosternites IV-VII with single seta m. Female genital papilla with 10+2+10 n.

Cerci (Fig. 10) singly segmented, symmetrical, with five distinct internal teeth, crooked; t3 larger than others; interval between t1-t2 as two times as t2-t3 and t3-t4; t2-t4 with shoulder, dorsal side with 9 M, 5 n and 5 m, ventral side with7 M, 3 n and 3 m; each cercus with 7–8 evaporation plates.

**Table 2.** Chaetotaxy of dorsal side of body in adult *Parajapyx pauliani*

segments		m	M				n
			ma	mp	la	lp	
Pronotum		6+6	1+1	1+1	2+2	1+1	6+6
Mesonotum	Prescutum	6+6					1+1
	Scutum	3+3	1+1	1+1	2+2	1+1	(8-13)+(8-12)
Metanotum	Prescutum	7+7					2+2
	Scutum	3+3	1+1	1+1	2+2	1+1	(13-17)+(10-16)
Abd. I	Prescutum	3+3					2+2
	Scutum	5+5	1+1	1+1	2+2	1+1	(10-14)+(9-14)
II	Prescutum	4+4					2+2
	Scutum	6+6	1+1	1+1	4+4	2+2	(11-17)+(11-17)
III	Prescutum	4+4					2+2
	Scutum	7+7	1+1	1+1	4+4	2+2	(14-17)+(11+17)
IV	Prescutum	4+4					2+2
	Scutum	7+7	1+1	1+1	4+4	2+2	(11-17)+(11-18)
V	Prescutum	4+4					2+2
	Scutum	7+7	1+1	1+1	4+4	2+2	(12-17)+(13-18)
VI	Prescutum	4+4					2+2
	Scutum	7+7	1+1	1+1	4+4	2+2	(12-14)+(10-16)
VII	Prescutum	4+4					2+2
	Scutum	8+8	1+1	1+1	4+4	2+2	(9-14)+(9-14)
VIII	Scutum	12+12	1+1	1+1	4+4	2+2	(9-10)+2(3)+(7-13)
IX	Scutum	7+7	3+3				2+1+2
X	Scutum	4+4	6+1+6				(6-8)+(6-8)

**Table 3.** Chaetotaxy of ventral side of body in adult *Parajapyx pauliani*

**segments**		**m**	**M**	**n**
Prosternum	Anterior lobe	3+7+3	2+2	1+1
	Middle lobe	7+7	2+2	2+2
	Posterior lobe	6+6	1+1	2(3)+1+2(3)
Mesosternum	Anterior lobe	3+3	2+2	2+2
	Middle lobe	7+7	4+4	2+2+2
	Posterior lobe	6+6	3+3	3+2+3
Metasternum	Anterior lobe	4+4	2+2	2+1+2
	Middle lobe	5+5	4+4	3+2+3
	Posterior lobe	5+5	3+3	3+2+3
Abd. I	Prescutum	2+2		5+5
	Scutum	5+5	10+10	(10-18)+2(3)+(11-18)
II	Prescutum	4+4		(5-7)+1+(5-7)
	Scutum	5+5	12+12	(8-11)+2(3)+(8-11)
III	Prescutum	4+4		6(7)+1+6(7)
	Scutum	5+5	12+12	(8-9)+2(3)+(8-11)
IV	Prescutum	4+4		6(7)+1+6(7)
	Scutum	5+5	12+12	(8-11)+2(3)+(8-11)
V	Prescutum	4+4		(5-7)+1+(5-7)
	Scutum	5+5	12+12	(7-12)+2+(7-10)
VI	Prescutum	4+4		6+1+6
	Scutum	5+5	12+12	(8-12)+2+(7-10)
VII	Prescutum	4+4		5+1+5
	Scutum	5+5	12+12	(9-10)+2+(6-9)
VIII	Scutum	4+4	2+2	(4-5)+(4-5)
IX	Scutum	4+4	2+2	3+3
X	Scutum	4+4	6+6	3+1+3

#### Distribution.

So far, the species is known only from two localities: Hainan, China and Madagascar.

#### Remarks.

*Parajapyx pauliani* is characterized by the antenna with 21 segments, nota each with 5+5 M setae and numerous normal setae, urotergites II-VII each with 8+8 M setae and numerous normal setae, and prescutum of urotergites II-V each with 2+2 normal setae. Ithas more normal setae than in other congeners. The numbers of M and m setae are relatively stable, but the numbers of normal setae are quite variable in different individuals.

##### Littoral habitat of *Parajapyx pauliani*

**Figures 18–20.**
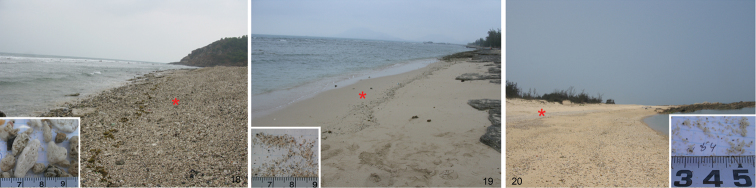
Habitats of *Parajapyx pauliani* in Hainan (S China). **18** shingly beach of Ximaozhou Island, inset shows the size of stone **19** sand beach of Ximaozhou Island, inset show the size of sand granules **20** sand beach of Qizi Bay, inset show the size of sand granules ✱ indicates the sample site.

Three intertidal locations where *Parajapyx pauliani* was found are shown in Figs 18–20. All habitats are positioned lower than supralittoral, devoid of halophytes, and are directly influenced by sea water. The animal lives in shingly or sand beaches (Figs 18–20), between particles of different size: from 9 mm (with the whole variation from 5 to 16 mm, n=100) to 1.5 mm (1.0–2.3 mm, n=150) in diameter. *Parajapyx pauliani* appears to be a dipluran member of a genuine littoral community and is often associated with collembolan species like *Yuukianura* sp., *Isotogastrura trichaetosa* Potapov et al. 2011, *Thalassaphorura* sp., *Oudemansia* sp., *Acherontiella* sp., *Archisotoma* sp.

**Figure 21.**
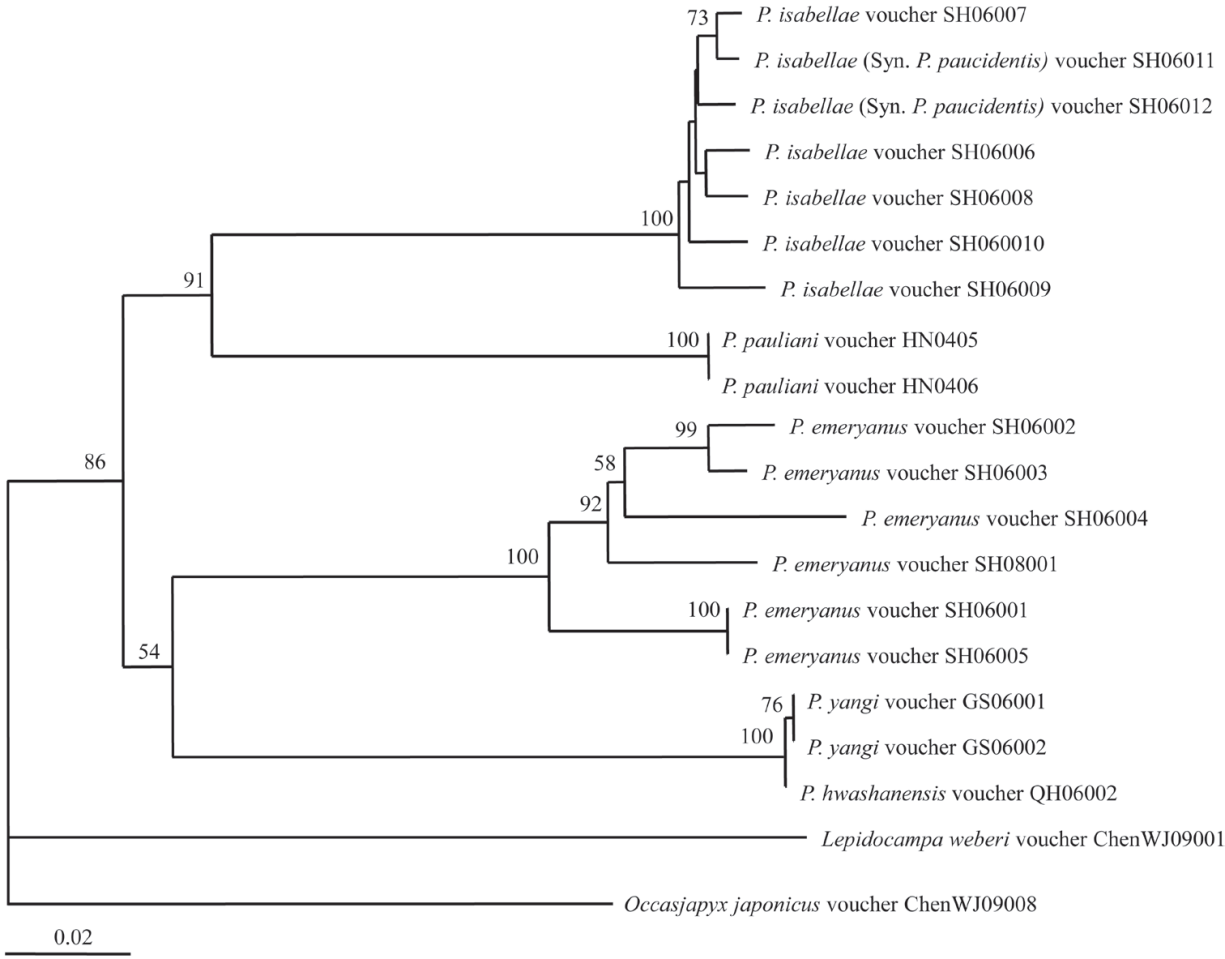
Neighbour-joining tree (p-distance, Bootstrap 1000 replicates) of Chinese *Parajapyx* inferred from COI gene sequences. Numbers on the nodes show the bootstrap values (> 50%).

##### The DNA barcoding

The DNA barcoding of 18 individuals from five *Parajapyx* species from China were sequenced, and deposited in GenBank (the accession numbers showed in Table 1). The genetic divergence between individuals of the same species is 1.9% in average, with span 1.5–5.3%, and it is 19.1% in average, with span 16.3–21.3% between different *Parajapyx* species.

The Neighbour-joining tree was constructed based on the barcoding sequences (Fig. 21). *Parajapyx pauliani* is clustered with *Parajapyx isabellae*. *Parajapyx isabellae* and *Parajapyx emeryanus* are valid species respectively well supported by barcoding analyses. Two individuals of *Parajapyx isabellae* (Syn. *Parajapyx paucidentis*) (teeth absent on the cerci) clustered together with five individuals of *Parajapyx isabellae* (teeth present on the cerci). The genetic divergence between *Parajapyx isabellae* (Syn. *Parajapyx paucidentis*) and *Parajapyx isabellae* is only 1.7% in average (with span 0.8–2.6%). In addition, individuals of *Parajapyx yangi* and *Parajapyx hwashanensis* clustered together with high support value, and the genetic divergence between them is low (0.2%).

## Discussion and conclusion

### Littoral records of *Parajapyx*

This is the first record of littoral dipluran in China. When *Parajapyx pauliani* was first found in intertidal zone in 1959, Pagés supposed that it is “purely fortuitous, and the single specimen collected was, in fact, might be pulled far away from its normal habitat by runoff” (Pagés 1959). Our records confirm the habitat of the species where it can live in narrow passages between sand particles due to slender and long body.

Numerous normal setae on body of *Parajapyx pauliani* are shared with *Parajapyx botosaneanui* Pagés, 1975, described from intertidal zone of Caribbean coast of Cuba (Pagés 1975). The two species can be readly distinguished by the number of the segments of antenna (21 in *Parajapyx pauliani* vs. 19 in *Parajapyx botosaneanui*). More dense setaceous covering probably protects the littoral species of *Parajapyx* against the periodical contact with salt water. Three other *Parajapyx* species *Parajapyx gerlachi*, *Parajapyx isabellae*, and *Parajapyx (G.) brasilianus* were also recorded in intertidal localities (Pagés 1967).

### Barcoding analysis

The DNA barcodes have been widely used in identification of microarthropod species, for instance, collembolans (Hebert et al. 2003, Hogg and Hebert 2004). To our knowledge, this is the first report on DNA barcodes of Diplura, which proved to be useful for dipluran identification. Our analyses confirmed the synonymy of *Parajapyx paucidentis* and *Parajapyx isabellae* proposed by Pagés (1998) and Luan et al. (2007). These species differed only by teeth in cerci, absent vs. present. The genetic divergence between *Parajapyx paucidentis* and *Parajapyx isabellae* is 1.7% in average (with span 0.8–2.6%), which is exactly in the span of the divergence between individuals of the same species.

The formal morphological difference in second problematic couple, *Parajapyx yangi* and *Parajapyx hwashanensis*, is the number of teeth on the cerci: the former species has four teeth, while the latter has five. Our DNA barcoding data showed only one nucleotide difference between examined individuals of *Parajapyx yangi* and *Parajapyx hwashanensis*. All individuals, identified formally by us as *Parajapyx yangi* were, however, immature that indicated the possible age nature of this differences. The type materials of the two species call for study to make the final conclusions.

## Supplementary Material

XML Treatment for Parajapyx pauliani
